# Loss of Caveolin-1 Is Associated with a Decrease in Beta Cell Death in Mice on a High Fat Diet

**DOI:** 10.3390/ijms21155225

**Published:** 2020-07-23

**Authors:** Paloma Lillo Urzúa, Olinda Núñez Murillo, Mauricio Castro-Sepúlveda, María A. Torres-Quintana, Álvaro Lladser Caldera, Andrew F. G. Quest, Carolina Espinoza Robles, Paola Llanos Vidal, Sergio Wehinger

**Affiliations:** 1 Departamento de Ciencias Biológicas, Facultad de Ciencias de la Vida, Universidad Andres Bello, Santiago 8370146, Chile; paloma.mlu@gmail.com; 2 Escuela de Microbiología, Universidad Nacional Autónoma de Honduras, Tegucigalpa 11101, Honduras; olindaivethnunez@gmail.com; 3 Escuela de Kinesiología, Facultad de Medicina, Universidad Finis Terrae, Santiago 7501015, Chile; mcastro@finis.cl; 4 Departamento de Patología y Medicina Oral, Facultad de Odontología, Universidad de Chile, Santiago 8380491, Chile; Mangelicatorres@odontologia.uchile.cl; 5 Laboratory of Immunoncology, Fundación Ciencia & Vida, Santiago 7780272, Chile; alladser@cienciavida.org; 6 Facultad de Medicina y Ciencia, Universidad San Sebastián, Santiago 7511055, Chile; 7 Laboratory of Cellular Communication, Program of Cell and Molecular Biology, Faculty of Medicine, Center for studies on Exercise, Metabolism and Cancer (CEMC), Institute of Biomedical Sciences (ICBM), University of Chile, Santiago 8380453, Chile; aquest@med.uchile.cl; 8 Thrombosis Research Center, Medical Technology School, Department of Clinical Biochemistry and Immunohematology, Faculty of Health Sciences, Universidad de Talca, Talca 3460000, Chile; carespinoza@utalca.cl; 9 Institute for Research in Dental Sciences, Facultad de Odontología, Center for Studies on Exercise, Metabolism and Cancer (CEMC), Universidad de Chile, Santiago 8380544, Chile; 10 Thrombosis Research Center, Center for Studies on Exercise, Metabolism and Cancer (CEMC), Medical Technology School, Department of Clinical Biochemistry and Immunohematology, Faculty of Health Sciences, Universidad de Talca, Talca 3481118, Chile

**Keywords:** ERK activity, caveolin-1, high fat diet, insulin resistance, diabetes

## Abstract

Elevated free fatty acids (FFAs) impair beta cell function and reduce beta cell mass as a consequence of the lipotoxicity that occurs in type 2 diabetes (T2D). We previously reported that the membrane protein caveolin-1 (CAV1) sensitizes to palmitate-induced apoptosis in the beta pancreatic cell line MIN6. Thus, our hypothesis was that CAV1 knock-out (CAV1 KO) mice subjected to a high fat diet (HFD) should suffer less damage to beta cells than wild type (WT) mice. Here, we evaluated the in vivo response of beta cells in the pancreatic islets of 8-week-old C57Bl/6J CAV1 KO mice subjected to a control diet (CD, 14% kcal fat) or a HFD (60% kcal fat) for 12 weeks. We observed that CAV1 KO mice were resistant to weight gain when on HFD, although they had high serum cholesterol and FFA levels, impaired glucose tolerance and were insulin resistant. Some of these alterations were also observed in mice on CD. Interestingly, KO mice fed with HFD showed an adaptive response of the pancreatic beta cells and exhibited a significant decrease in beta cell apoptosis in their islets compared to WT mice. These in vivo results suggest that although the CAV1 KO mice are metabolically unhealthy, they adapt better to a HFD than WT mice. To shed light on the possible signaling pathway(s) involved, MIN6 murine beta cells expressing (MIN6 CAV) or not expressing (MIN6 Mock) CAV1 were incubated with the saturated fatty acid palmitate in the presence of mitogen-activated protein kinase inhibitors. Western blot analysis revealed that CAV1 enhanced palmitate-induced JNK, p38 and ERK phosphorylation in MIN6 CAV1 cells. Moreover, all the MAPK inhibitors partially restored MIN6 viability, but the effect was most notable with the ERK inhibitor. In conclusion, our results suggest that CAV1 KO mice adapted better to a HFD despite their altered metabolic state and that this may at least in part be due to reduced beta cell damage. Moreover, they indicate that the ability of CAV1 to increase sensitivity to FFAs may be mediated by MAPK and particularly ERK activation.

## 1. Introduction

The western lifestyle, characterized by sedentarism and fat-rich diets, has positioned obesity and its complications amongst the most important public health problems worldwide. Among the diseases strongly associated with overweight and obesity, type 2 diabetes (T2D) is of notable concern [1]. The pathogenesis of T2D is complex and involves, on the one hand, hereditary genetic factors and, on the other hand, external environmental factors (sedentarism, high fat diet, etc.). Two main components are thought to converge in the development of T2D, namely insulin resistance and beta cell dysfunction and death [2]. One of the principal factors involved in beta cell dysfunction and death is the lipotoxicity induced by free fatty acids. Lipotoxicity refers to an excessive accumulation of lipids that leads to metabolic abnormalities in several organs, such as the liver, muscle, heart and pancreatic islets. Many studies are available addressing the phenomenon of insulin resistance due to lipotoxicity, and although the contribution to beta cell dysfunction is also well established, the underlying mechanisms in this case are less well understood. It should be noted that insulin resistance by itself is not sufficient to explain the onset of T2D. In fact, beta cells respond to chronic nutrient excess and to ensuing insulin resistance by increasing insulin secretion in order to maintain normal levels of glucose [3,4]. T2D develops only in individuals that are unable to sustain this compensatory response, and T2D development coincides with a progressive loss of pancreatic beta cell mass by apoptosis [5]; however, the factors involved are still a matter of debate.

High concentrations of free fatty acids are associated with obesity and insulin resistance in skeletal muscle and liver [6,7]. Moreover, high levels of free fatty acids are a risk factor for the development of T2D and previous reports show that fatty acids contribute directly to dysfunction due to lipotoxicity and cell death by apoptosis in beta pancreatic cells [8]. Several mechanisms have been proposed to explain how palmitate induces beta cell dysfunction and death. Indeed, we recently reported that ROS (reactive oxygen species) production following the exposure of MIN6 murine beta cells to high concentrations of palmitate is the consequence of ceramide synthesis, which in turn damages mitochondria. Importantly, we observed that the presence of the scaffolding protein caveolin-1 (CAV1) increases in vitro cell death in these cells [9]. However, whether the absence of CAV1 protects beta cells in a CAV1 knock-out (CAV1 KO) animal model subjected to a high fat diet remained to be determined.

CAV1 is a membrane-bound scaffolding protein that belongs to a family of proteins with three members: CAV1, CAV2 and CAV3. The best-known role of CAV1 is as a crucial component required for the genesis of caveolae, flask-like invaginations of the plasma membrane, which are important in vesicular trafficking, cholesterol transport and especially transcytosis in epithelial and endothelial cells [10]. Importantly, CAV1 function is not restricted to vesicular trafficking and transport, since this protein also participates in the regulation of diverse signaling cascades, serving as a scaffolding protein. Indeed, CAV1 possesses a scaffolding domain (CSD) containing nearly 20 aminoacids [11] that is implicated in interactions with a large number of signaling proteins, including G-protein coupled receptors, Src family kinases, adenylyl cyclases, ion channels, endothelial oxide nitric synthase and protein kinase A, among others [12]. Additionally, CAV1 has been associated with sensitization to cell death and induction of apoptosis [13]. CAV1 is normally present in beta cells, where it participates in the regulation of insulin secretion [14,15], and recently, CAV1 was also shown to regulate insulin receptor internalization and ERK activation in human and mouse beta cells [16], implicating CAV1 as a relevant player in beta cell physiology. However, CAV1 also seems to play a role in beta cell death in response to oxidative stress induced by high levels of palmitate in vitro, as we reported for the first time [9]. Thus, we hypothesized that in an animal model lacking CAV1 subjected to a high fat diet (HFD), beta cell damage should be reduced compared to that observed in wild type animals.

Mitogen-activated protein kinases (MAPKs) are serine-threonine kinases that participate in essential cell signaling pathways associated with cellular functions ranging from promoting cell survival to enhancing cell death. In particular, three kinases in the MAPK pathways have been associated with responses to cellular stress, namely c-jun NH2-terminal kinase (JNK), p38 and ERK1/2. Whether these responses protect against cell demise or, conversely, promote stress-induced cell death, appears to be cell context-dependent [17]. Activation of these MAPK pathways in response to cellular stress has been implicated in apoptotic-signaling responsible for beta cell loss [18,19,20,21,22,23]. CAV1 has been linked to the regulation of MAPKs by either activating or inhibiting these kinases, with different consequences concerning cell viability. For instance, it has been reported that CAV1-null mice are resistant to hyperoxia-induced lung injury through p38 inhibition [24]. On the other hand, CAV1 can activate MAPK pathways in response to stress and can trigger apoptosis. Specifically, CAV1 phosphorylation on tyrosine-14 enhances sensitivity to the anticancer drug paclitaxel through JNK activation [25].

Here, our objective was to evaluate the in vivo response of beta cells in the pancreatic islets of a mouse model, with a homozygous germ-line deletion of the CAV1 gene when subjected to a long-term (12-week) high fat diet (HFD). In addition, we compared the metabolic condition and glucose management of such KO mice with the responses in wild type (WT) mice. We worked with 8-week-old C57Bl/6J mice, because at this age the animals show notable beta cell responses [26]. To study the possible implication of MAPK association in the CAV1-induced sensitization of beta cells to damage by lipotoxicity, we used the well-characterized murine beta cell line MIN6, expressing or not expressing CAV1. We observed a lower percentage of beta cell apoptosis in islets of KO compared to WT mice on HFD. On the other hand, in the MIN6 murine beta cell line, CAV1 enhanced palmitate-induced activation of the MAPKs, ERK, JNK and p38. However, only ERK activation appeared to be relevant to CAV1-induced sensitization to palmitate, as evidenced using pharmacological inhibitors.

## 2. Results

### 2.1. Body Weight Visceral Fat and Blood Lipids

**Figure 1:**
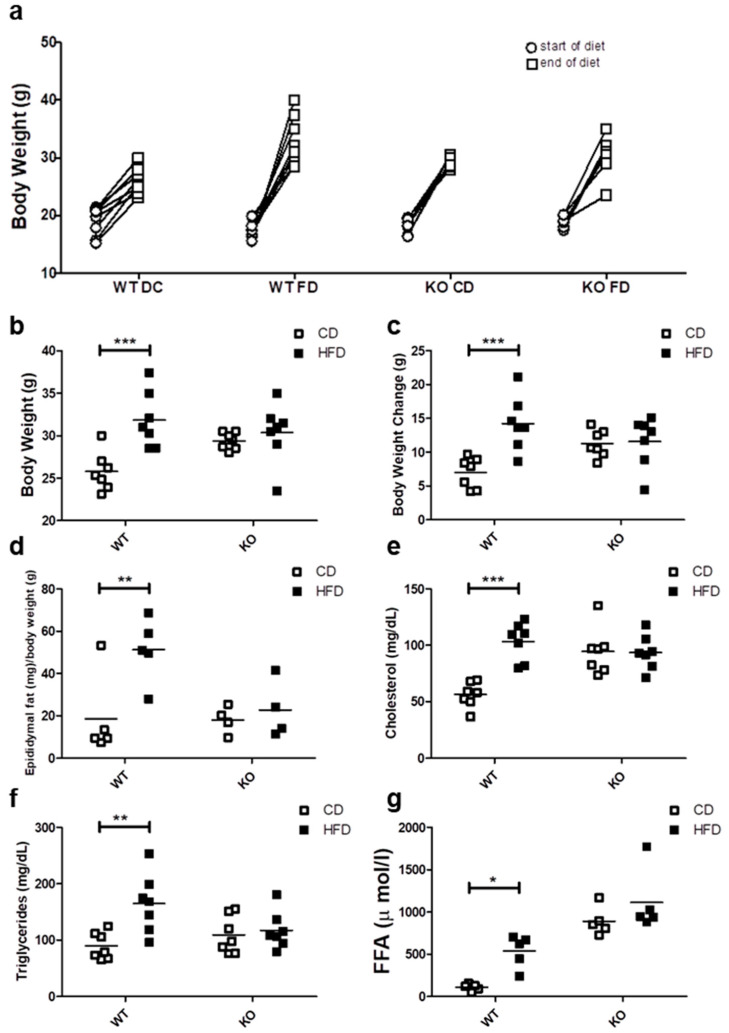
Metabolic characterization of male C57BL6J wild type (WT) and C57BL6J caveolin-1 knock-out (CAV1 KO) mice. (**a**) The body weight at the beginning (circles) and the end (squares) of the 12 weeks for mice on control diet (CD) or high fat diet (HFD) (*n* = 7). (**b**) Final body weight at the end of diets (*n* = 7). (**c**) Body weight changes between the beginning and the end of diets (*n* = 7). (**d**) The animals were sacrificed to determine the epididymal fat (*n* = 5). Serum samples were taken in order to evaluate the blood levels of (**e**) total cholesterol (*n* = 7), (**f**) triglycerides (*n* = 7) and (**g**) free fatty acids (FFA, *n* = 5). Mice were maintained for 6 h in fast conditions before the blood samples were taken. *p* values were calculated by two-way ANOVA with a Bonferroni post-test (* *p* < 0.05, ** *p* < 0.01 and *** *p* < 0.001). Data are presented as individual data points with their respective means.

After 12 weeks of control (CD) or high fat diet (HFD), progressive weight gain was observed in all mice, because they continued to grow due to their age (Figure 1a). However only the WT mice fed on HFD showed a significant weight gain as compared with WT on CD, reaching an average body weight of 15.5 ± 5.2 vs. 7.02 ± 2.1 g (*p* < 0.001), respectively. In contrast, CAV1 KO mice were resistant to increases in weight after being on HFD, with values similar to KO mice fed with CD (HFD/11.26 ± 2.0 vs. CD/11.58 ± 3.7 g). A notable and significant increase in the visceral adipose epididymal tissue was observed in WT mice on the HFD (CD/18.6 ± 19.4 vs. HFD/51.2 ± 15.1 mg/g; *p* < 0.05; Figure 1d), while there was no significant increase in CAV1 KO mice (CD/18.1 ± 6.5 vs. HFD/22.8 ± 13.6 mg/g).

Next, we evaluated the effect of HFD on blood lipid levels. As shown in Figure 1e, cholesterol levels tended to be higher in KO mice, but they were significantly elevated only in WT mice on HFD (CD/56.3 ± 11.2 vs. HFD/103.4 ± 16.8 mg/dL; *p* < 0.001). Moreover, HFD increased the triglyceride levels only in WT mice (CD/89.6 ± 24.0 mg/dL vs. HFD/165.0 ± 52.3 mg/dL; *p* < 0.01; Figure 1f). Mice lacking CAV1 had higher free fatty acid (FFA) levels (CD/890.0 ± 167.3 µmol/L and /1112.4 ± 372.8 µmol/L), but the HFD significantly increased FFA levels only in WT mice (CD/109.4 ± 38.9 vs. HFD/537.2 ± 194.0; *p* < 0.05; Figure 1g). Taken together, these results suggest that KO mice are resistant to weight gain and to the increases in blood lipid levels when put on a HFD, but they display an abnormal lipid metabolism even when on CD.

### 2.2. Glucose Insulin and Oxidative Stress

**Figure 2:**
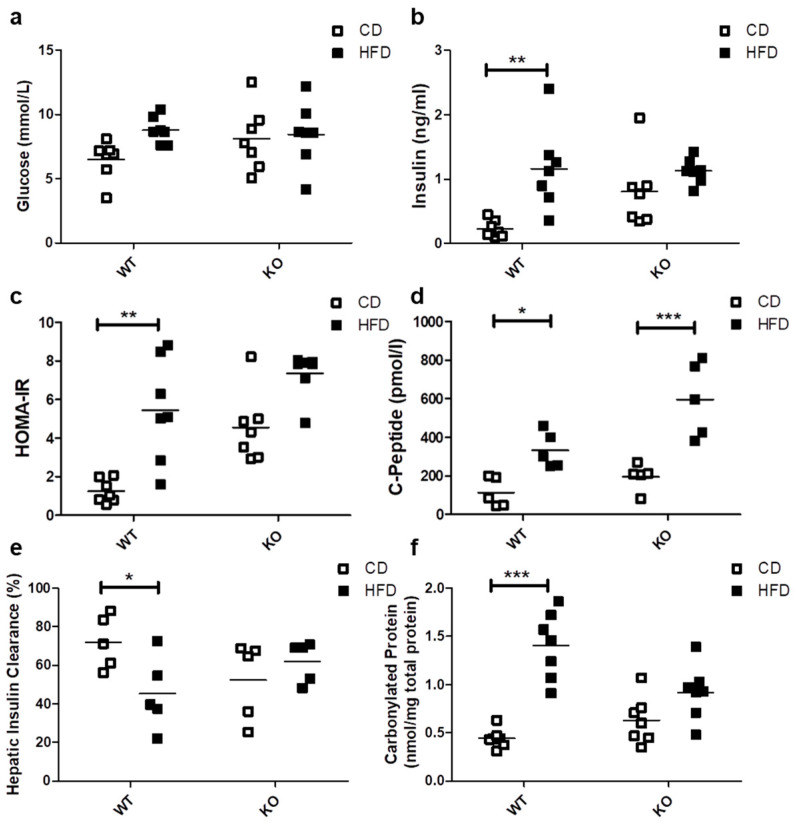
Glucose management, Insulin and C-peptide levels, HOMA-IR, hepatic insulin clearance and oxidative stress in WT and KO mice subjected to CD or HFD. After 12 weeks of diet, blood samples were taken in order to evaluate the serum levels of (**a**) basal glucose (*n* = 7), (**b**) basal insulin (*n* = 7), (**c**) HOMA-IR (*n* = 7), (**d**) basal C-peptide (*n* = 5), (**e**) hepatic insulin clearance and (**f**) carbonylated proteins (*n* = 7). HOMA-IR (Homeostatic Model Assessment of Insulin Resistance) adjusted for mice (*n* = 7) were calculated from basal glucose and insulin levels. Hepatic insulin clearance was calculated from the basal insulin/C-peptide ratio (*n* = 5). Mice were maintained for 6 h in fasting conditions before blood samples were taken. *p* values were calculated by two-way ANOVA with a Bonferroni post-test (* *p* < 0.05, ** *p* < 0.01, *** *p* < 0.001). Data are presented as individual data points with their respective means.

The HFD did not induce significant differences in basal blood glucose levels in either WT (CD/6.51 ± 1.50 vs. HFD/8.79 ± 1.04 mmol/L) or CAV1 KO mice (CD/8.11 ± 2.49 vs. HFD/8.45 ± 2.49 mmol/L; Figure 2a). A significant increase in basal insulin levels was observed only in WT mice subjected to HFD (CD/0.23 ± 0.13 vs. HFD/1.16 ± 0.65 ng/mL; *p* < 0.01; Figure 2b). However, the basal insulin levels in CAV1 KO mice were higher compared with WT mice on CD (WT/0.23 ± 0.13 vs. KO/0.81 ± 0.56 ng/mL; *p* < 0.05). Consequently, the adjusted HOMA-IR (Homeostatic Model Assessment of Insulin Resistance) index increased significantly in WT mice on HFD (CD/1.25 ± 0.61 vs. HFD/5.45 ± 2.68; *p* < 0.01; Figure 2c). For CAV1 KO mice, higher levels of adjusted HOMA-IR were observed in those on CD, while for mice on HFD, there was an increase, but it was not statistically significant (CD/4.55 ± 1.82 vs. HFD/7.35 ± 1.17). C-peptide levels were significantly augmented in WT mice on HFD (CD/114.0 ± 76.5 vs. HFD/333.2 ± 92.9 pmol/L; *p* < 0.05; Figure 2d). This effect was even more pronounced in CAV1 KO mice (CD/174.6 ± 80.1 vs. HFD/567.7 ± 187.0 pmol/L; *p* < 0.001). The HFD induced a significant reduction in hepatic insulin clearance only in WT mice (CD/72.1% ± 13.8% vs. HFD/45.3% ± 19.1%; *p* < 0.05), while for CAV1 KO mice, changes in the hepatic insulin clearance were not significant (CD/52.5% ± 20.3% vs. HFD/62.1% ± 10.6%), but their values tended to be lower than WT on CD. Taken together, these results suggest that CAV1 KO mice are insulin resistant, even when on CD.

There is a close association between lipotoxicity and oxidative stress [9,27]. With this in mind, we evaluated the oxidative status by measuring carbonylated proteins in mice after being on either CD or HFD. The HFD notably increased carbonylated protein levels only in WT mice (CD/0.44 ± 0.10 vs. HFD/1.40 ± 0.35 nmol/mg total protein; *p* < 0.001; Figure 2e). For the CAV1 KO mice on HFD, differences in the levels of carbonylated proteins compared to those on CD were not significant (*p* = 0.0842). However, they tended to be lower in CAV1 KO compared with WT mice on HFD (WT/1.40 ± 0.35 vs. KO/0.92 ± 0.28 nmol/mg total protein), with an interaction between diet and CAV1 condition with a p value of 0.0021. This may be taken to suggest that CAV1 expression promotes oxidative stress in mice on HFD.

### 2.3. Intraperitoneal Glucose Tolerance Test (IPGTT)

**Figure 3:**
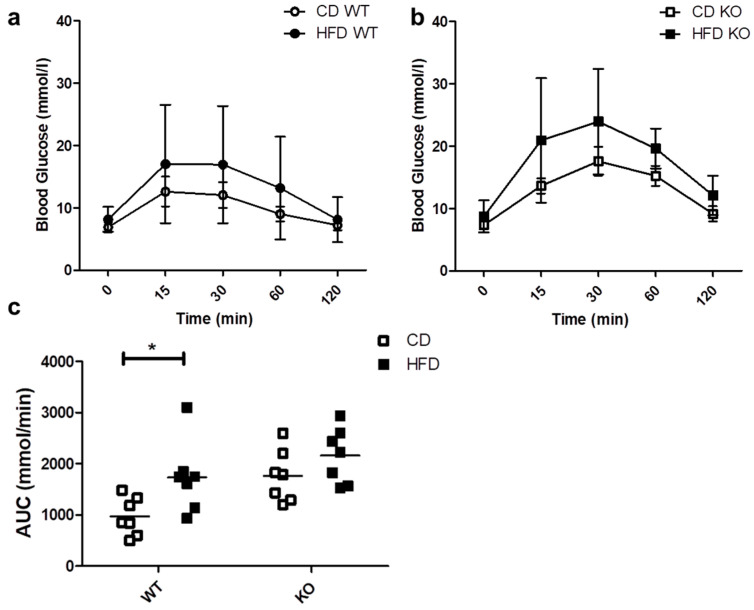
IPGTT in WT and KO mice after 12 weeks of CD or HFD. Mice were subjected to a 6-h fasting period, and then a sample of caudal capillary blood was taken (basal). Then, mice were subjected to an intraperitoneal glucose load (2 g/Kg), and a glycemia test was taken in the same way at different time points, up to 120 min. The response kinetics are shown for WT mice in (**a**) and KO mice in (**b**). The area under the curve (AUC) in (**c**) was calculated and plotted for each group. *p* values were calculated by two-way ANOVA with a Bonferroni post-test (* *p* < 0.05). The data shown are the averages from results obtained in seven WT and seven KO mice per group. Data are presented as mean ± SD in (**a**,**b**) and as individual data points with their respective means in (**c**).

We evaluated the ability of mice to respond to a glucose load through an IPGTT. As shown in Figure 3a,b, the blood glucose levels after 30 min tended to be higher both in WT and KO mice on HFD as compared to CD, but the differences were not statistically significant. As shown in Figure 3c, HFD induced a significant increase in the area under the curve (AUC) only in WT (CD/615.3 ± 258.4 vs. HFD/1201.5 ± 366.3 mmol/min; *p* < 0.05) and not in CAV1 KO mice (CD/988.0 ± 190.9 vs. HFD/1190.4 ± 347.9 mmol/min; *p* = 0.2021). However, for mice on CD, the AUC was higher than in WT mice (WT/615.3 ± 258.4 vs. KO/988.0 ± 190.9 mmol/min). Therefore, glucose tolerance is impaired in CAV1 KO mice in a manner independent of the diet.

### 2.4. Study of Islet Morphology and Proliferation

**Figure 4:**
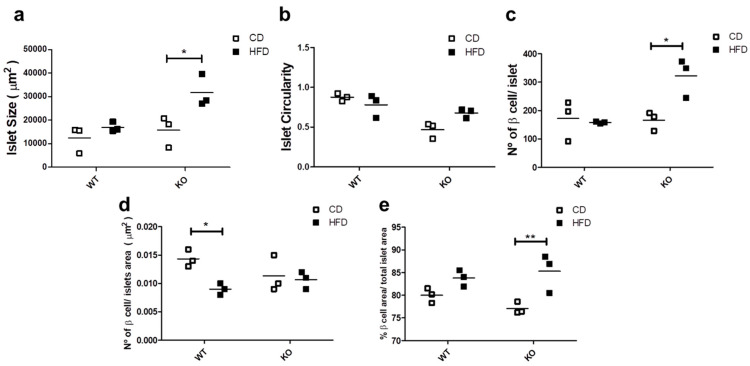
Study of islet morphology. After 12 weeks on CD or HFD, the pancreas was extracted, and 4 mm pancreas sections were stained with DAPI (4’,6-Diamidino-2-Phenylindole (double stranded DNA staining) and insulin-specific antibodies to identify the islets. In (**a**) the islet size, (**b**) the islet circularity, (**c**) the number of beta cells per islet, (**d**) the beta cell density in the islet and (**e**) the total beta cell area (%) per total islet area are shown. The results are representative of at least three sections per animal, with a total of at least three animals for each WT and KO group. *p* values were calculated by two-way ANOVA with a Bonferroni post-test (* *p* < 0.05, ** *p* < 0.01). Data are presented as individual data points with their respective means.

Since glucose homeostasis and insulin demands affect the islet morphology and cell number [28], we evaluated the morphological aspects of the islets in WT and CAV1 KO mice after 12 weeks on CD or HFD. As shown in Figure 4a, islet size increased significantly with HFD only for KO mice (CD/15,770 ± 7588 vs. HFD/31,700 ± 8010 µm^2^; *p* < 0.05; Figure 4a). The HFD had no effect on the islet circularity in either group of mice (Figure 4b). The number of beta cells per islet was significantly higher in KO mice on HFD (WT/157.7 ± 4.06 vs. KO/322 ± 79.22 beta cells/islet; *p* < 0.05; Figure 4c). Beta cell density was significantly lower in the islets of WT mice on HFD than those on CD (WT/0.0143 ± 0.0017 vs. KO/0.0090 ± 0.0011, beta cell/islet area in µm^2^; *p* < 0.05; Figure 4d). Finally, the HFD induced a significant increase in the area occupied by beta cells per islet, but only in KO mice (CD/77.1% ± 1.22% vs. HFD/85.3% ± 3.46%; *p* < 0.01; Figure 4e). Taken together, these results point towards the existence of differences in the effects on islet morphology induced by HFD in KO mice. Specifically, the islet size, the number of beta cells per islet and the area occupied by beta cells increased, while the islet circularity and the beta cell density were maintained. Alternatively, we only observed a decrease in the beta cell density in WT mice on HFD.

### 2.5. Study of In Situ Islet Cell Apoptosis

**Figure 5:**
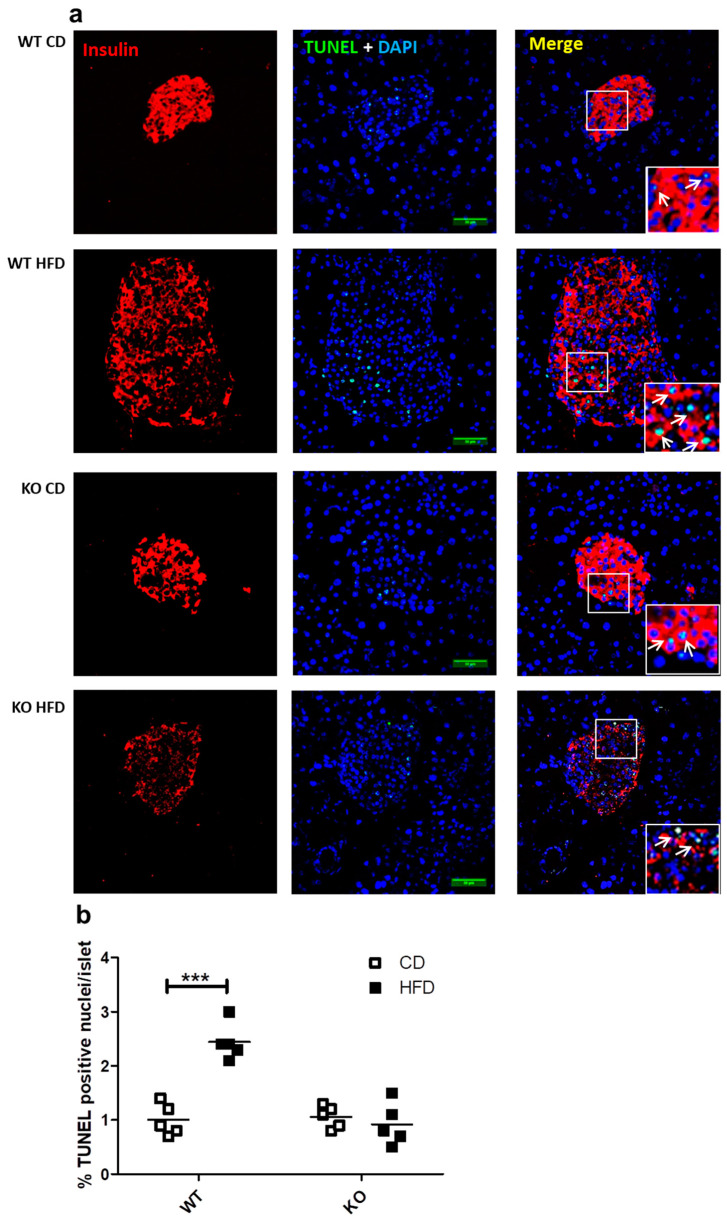
Study of apoptosis in situ in beta islets of WT and KO mice exposed to CD or HFD. Pancreas sections of 4 mm were stained with DAPI and immunostaining for insulin in order to identify the beta cells in islets, and apoptosis was determined by TUNEL (Terminal deoxynucleotidyl transferase dUTP nick end labeling) staining and analyzed by confocal microscopy. (**a**) White arrows indicate examples of TUNEL-positive nuclei within the islets. Scale bar represents 50 µm. Percentage of apoptotic TUNEL+ nuclei per islet is quantified in (**b**) The results are representative of at least three sections per animal, with a total of five animals for each WT and KO group. *p* values were calculated by two-way ANOVA with a Bonferroni post-test, with *** *p* < 0.001. Data are shown as individual data points with their respective means.

We previously reported that the presence of CAV1 promotes palmitate-induced cell death in the beta cell line MIN6 in vitro [9]. In the present study, we evaluated whether the number of apoptotic cells in the beta islets of mice expressing or not expressing CAV1 varied when on HFD. As shown in Figure 5a,b, HFD induced a significant increase in the number of TUNEL+ (Terminal deoxynucleotidyl transferase dUTP nick end labeling) apoptotic cells only in WT mice (CD/1.02% ± 0.27% vs. HFD/2.44% ± 0.34%; *p* < 0.001). For the CAV1 KO mice on HFD, no changes in the percentage of TUNEL+ cells were apparent (CD/1.06% ± 0.21% vs. HFD/0.92% ± 0.39%; *p* = 0.4985). These results indicate that CAV1 ablation tends to reduce beta cell apoptosis in the islets of mice on HFD for 12 weeks.

### 2.6. Role of MAPK Activation in Beta Cell Lipotoxicity In Vitro

**Figure 6:**
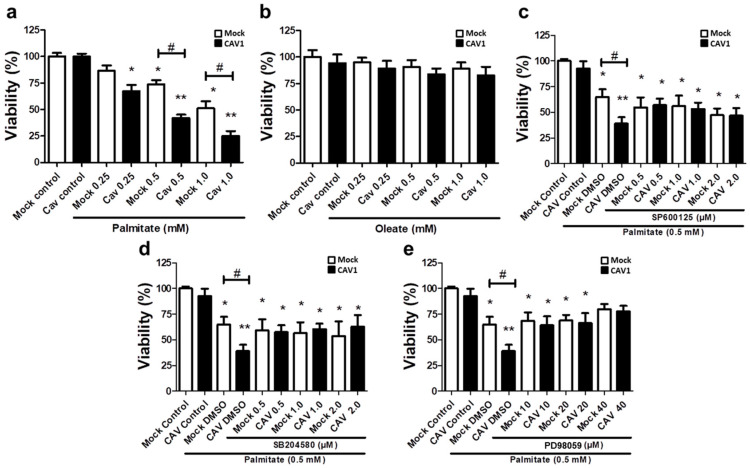
Role of MAPK in in vitro lipotoxicity in MIN6 cells in the presence or absence of CAV1. MIN6 mock and MIN6 CAV1 cells were incubated with different concentrations of FFA (**a**) palmitate or (**b**) oleate for 24 h, and viability was determined by the MTT (3-(4,5-dimethylthiazol-2-yl)-2,5-diphenyltetrazolium bromide) assay. (**c**–**e**): The cells were pre-incubated with MAPK inhibitors for 30 min and then with 0.5 mM palmitate for 24 h. After this, cell viability was determined by the MTT assay. The results are expressed as the percentage viability compared to control cells (only medium with DMSO) or cells pre-incubated with inhibitors. The results represent the mean ± SD of six independent experiments (* *p* < 0.05 and ** *p* < 0.01 with respect to control; # *p* < 0.05 mock vs. CAV1).

Bearing in mind the aforementioned results obtained in CAV1 KO mice, we next sought to better understand the cell signaling pathways involved in the CAV1-enhanced sensitivity of islet beta cells to apoptosis in vivo after 12 weeks on HFD. To this end, we evaluated the lipotoxic effects of high levels of free fatty acids in a murine beta pancreatic cell line expressing (MIN6 CAV1) or not expressing (MIN6 mock) the CAV1 protein. Alterations in MAPK signaling have been involved in a wide variety of diseases, including obesity and diabetes, as well as in palmitate-induced cell death [29,30,31]. Therefore, we explored the possibility that JNK, p38 and/or ERK 1/2 might be relevant to the susceptibility of beta cells to lipotoxicity in the presence of CAV1. We exposed MIN6 cells to different concentrations of the FFAs palmitate (saturated) or oleate (monounsaturated) for 24 h. We observed a significant decrease in beta cell viability in the presence of 0.25 mM of palmitate (Figure 6a) in CAV1-expressing MIN6 cells (67.2.0 ± 6.2; *p* < 0.05). For the MIN6 CAV1 cells, sensitivity to 0.5 mM palmitate was increased compared to MIN6 mock cells (viability mock/73.8% ± 3.8% vs. CAV1/41.9% ± 3.3%; *p* < 0.05) and 1.0 mM (viability mock/51.2% ± 6.6% vs. CAV1/24.9% ± 4.7 %; *p* < 0.05). We choose 0.5 mM palmitate for the following experiments because 1.0 mM did not significantly increase the difference between MIN6 mock and CAV1 cells. Notably, oleate did not induce a significant change in beta cell viability in vitro at any of the concentrations tested, in either cells expressing CAV1 or cells not expressing CAV1 (Figure 6b). To evaluate the possibility that MAPK activity was involved in the loss of viability induced by palmitate and, in particular, the increased sensitivity induced by CAV1, we incubated MIN mock and CAV1 cells with 0.5 mM palmitate in the presence or absence of the three MAPK inhibitors SP600125 (JNK), SB204580 (p38) or PD98059 (MEK/ERK). We observed that all three inhibitors diminished individually the difference in viability observed following palmitate incubation of MIN6 mock and CAV1 cells (Figure 6c–e). However, only the MEK/ERK inhibitor PD98059 induced an increase in MIN6 CAV1 cell viability compared with the controls (Figure 6d). These results with MAPK inhibitors suggest that ERK activity participates, at least in part, in palmitate-induced cytotoxicity.

**Figure 7:**
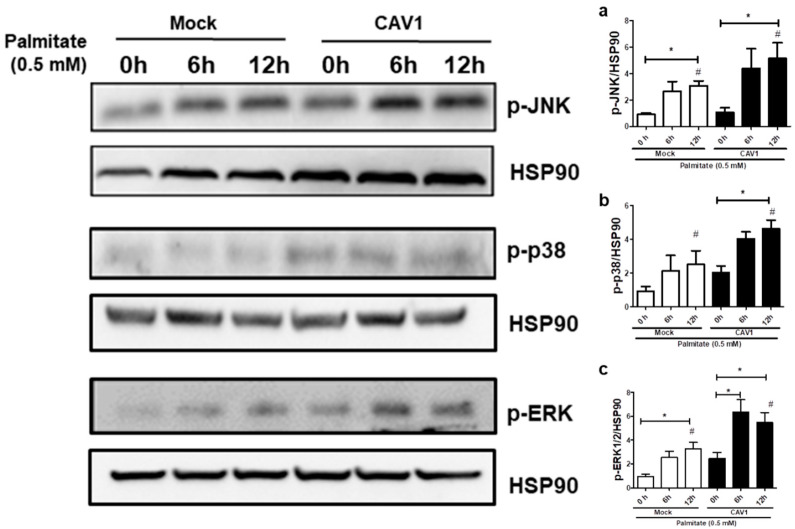
MAPK activation by palmitate in the presence or absence of CAV1. The MIN6 mock and CAV1 cells were exposed to 0.5 mM palmitate and collected at 0, 6 and 12 h for protein extraction and Western blot to evaluate the activation of phosphorylation of (**a**) JNK, (**b**) p38 and (**c**) ERK. The presence of HSP90 was displayed as a load control. The results are represented as the mean ± SD and are representative of three independent experiments. * *p* < 0.05; with respect to 0 h; # *p* < 0.05; mock vs. CAV1.

Next, to test if CAV1 modulates MAPK activation in beta cells exposed to palmitate, we evaluated MAPK phosphorylation levels in MIN6 cells exposed to palmitate in the presence or absence of CAV1. Incubation for 12 h with palmitate increased the phosphorylation (Figure 7) of JNK in MIN6 mock cells (3.08 ± 0.38 fold; *p* < 0.05 after 12 h with respect to the control 0 h; Figure 7a) and MIN6 CAV1 cells (5.15 ± 1.19 fold; *p* < 0.05 at 12 h with respect to the 0 h control; Figure 7a). Increased phosphorylation of p38 was also observed. However, this increase was only significant in CAV1-expressing cells (4.63 ± 0.50 fold; *p* < 0.05 at 12 h; Figure 7b). Palmitate activated ERK1/2 significantly at 12 h in MIN6 mock cells (3.27 ± 0.53 fold; *p* < 0.05; Figure 7c). However, in CAV1-expressing cells, increased phosphorylation was significant at 6 h (6.33 ± 1.06 fold; *p* < 0.05; Figure 7c) and at 12 h (5.47 ± 0.85; *p* < 0.05; Figure 7c) of incubation. Additionally, a significant increase in phosphorylation was observed in CAV1 compared to mock cells that were treated for 6 h with palmitate for JNK (mock/3.08 ± 0.38 vs. CAV1/5.15 ± 1.19 fold; *p* < 0.05; Figure 7a), p38 (mock/2.03 ± 0.40 vs. CAV1/4.63 ± 0.50 fold; *p* < 0.05; Figure 7b) and ERK1/2 (mock/3.27 ± 0.53 vs. CAV1/5.47 ± 0.85 fold; *p* < 0.05; Figure 7c). Taken together, these in vitro results suggest that the presence of CAV1 favors palmitate-induced MAPK activation and, in combination with the inhibitor data (see previous sections), that these changes are relevant to beta cell death.

## 3. Discussion

In our experiments, mice lacking CAV1 were resistant to weight gain and epididymal fat accumulation after three months on HFD (Figure 1a). Reduced weight gain after a HFD in CAV1 KO mice has been reported previously, and it has been associated with intestinal fatty acid absorption [32]. On the other hand, CAV1 is abundantly expressed in adipocytes, and the absence of this protein reportedly affects adipocyte homeostasis [33,34]. However, resistance to weight gain and/or visceral fat accumulation is not associated with an improved metabolic status, as our results indicate (Figure 1e–g). In an early study, Razani et al. found that CAV1 KO mice have hypertriglyceridemia and increased free fatty acids levels [33]. Additionally, CAV1 has been suggested to play an important role in promoting reverse cholesterol transport [35]. This could explain, at least in part, the elevated levels of total cholesterol found in KO mice (Figure 1e). On the other hand, the high levels of FFAs in mice lacking CAV1, as reported here, agree with the general concept that CAV1-null mice are lipodystrophic [36,37,38]. These elevated levels of free fatty acids can be attributed to altered function of adipose tissue and elevated lipolysis due to alterations in insulin responses [39,40].

With respect to glucose management, we observed a tendency towards increased fasting glycemia in CAV1 KO mice, although differences were not significant (Figure 2a). Based on the available literature, the lack of CAV1 in mice has been associated with poor adaptation to altered metabolic conditions; however, alterations in the levels of glycemia are not always present [41]. Significant hyperglycemia associated with enhanced gluconeogenesis and elevated lipolysis in CAV1 KO mice has been reported [38,42,43]. In our study, HFD induced more significant increases in the insulin levels and adjusted HOMA-IR values of WT compared to CAV1 KO mice (Figure 2c); however, elevated levels of insulin and adjusted HOMA-IR values were found in KO mice on CD. In fact, it has been reported that CAV1-null mice develop a compensatory response in the liver, which is triggered by defective mitochondrial metabolism in adipose tissue [38]. This suggests that although CAV1 KO mice have a poor metabolic status, they still adapt to a HFD.

The high levels of blood insulin and adjusted HOMA-IR in CAV1 KO mice on a HFD are in agreement with the augmented blood levels of C-peptide (Figure 2d), since C-peptide is secreted in equimolar concentrations with insulin, and increased levels of this peptide and insulin in the blood stream are indicative of insulin resistance [44,45]. Intriguingly, we observed in CAV1 KO mice that the increase in C-peptide levels was more significant than in WT mice, but this was not the case for insulin. This may relate to the differences in the half-life of the two polypeptides. C-peptide has a longer half-life than insulin in circulation (20–30 s vs. 3–5 min, respectively), and the liver metabolizes circulating insulin but not C-peptide, which is cleared by the kidney. Additionally, C-peptide is removed from the blood stream at a rate that is notably less variable and slower than insulin, so differences between insulin and C-peptide secretion have been reported [46,47]. Moreover, the CAV1 KO mice had higher insulin levels even when on CD. This could explain why we observed a significant increase in C-peptide but not in insulin in KO mice. Another possibility is that the difference could be due in part to the elevated removal of insulin in the liver or the reduced clearance of C-peptide in the kidneys of KO mice. In fact, the hepatic insulin clearance calculated in our experiments was higher in CAV1 KO mice than in WT mice on HFD, despite the fact that, for KO mice, hepatic insulin clearance was lower in those on CD (Figure 2e). Reduced hepatic insulin clearance is reportedly associated with insulin resistance and diet-induced obesity in rats [48] and with diabetic conditions in humans [49]. The significant decrease in the hepatic insulin clearance in WT mice on HFD that was observed in our experiments is consistent with these reports. Intriguingly, the HFD did not reduce hepatic insulin clearance in KO mice, as was also the case for other metabolic parameters evaluated in this study. In the same way, for CAV1 KO mice, diminished glucose tolerance was observed in those on CD, and the HFD did not significantly worsen this condition (Figure 3). In summary, these results suggest that CAV1 KO mice have an altered lipid and glucose metabolism with insulin resistance but that long-term HFD does not alter significantly this condition.

The oxidative stress associated with high levels of FFAs and glucose has been proposed to represent a major contributing factor to beta cell dysfunction and cell death in T2D [50]. We observed an increase in carbonylated proteins in the blood samples of animals on the HFD, but only in WT mice (Figure 2e). This result was unexpected given that an altered and pro-lipotoxic metabolism should increase oxidative stress, as reported for CAV1 KO mice [38]. However, we previously reported that in MIN6 beta cells, the presence of CAV1 had no significant effects on palmitate-induced oxidative stress in vitro [9].

In our experiments, we only observed a significant decrease in beta cell density in the islets of WT mice on HFD. In contrast, for the CAV1 KO mice, an increase in islet size, beta cell circularity and the area occupied by beta cells per islet was observed (Figure 4). This represents an islet response that may be expected due to the altered metabolic state of CAV1 KO mice. On the other hand, this could also suggest that CAV1 regulates the morphological response of the beta islets to a HFD. Indeed, the islets from KO mice on CD tended to be less circular than those of WT animals. The circularity is associated with smaller but functional islets, while bigger islets tend to be more elongated [51,52]. However, the heterogeneity in terms of the shapes and sizes of the islets can be seen even in a healthy pancreas, which makes it difficult to interpret the data and correlate morphological changes in islets with specific pathological conditions [51].

The HFD induced a significant increase in the TUNEL staining of beta cells in WT islets but not in CAV1 KO mice (Figure 5b). This finding is consistent with the general conclusion of our previous study, in which we observed that CAV1 presence sensitized cells to palmitate-induced cell death in vitro [9]. Here, it is important to note that even in CD conditions, no significant increases in the number of apoptotic cells in the CAV1 KO islets were detected, despite their otherwise lipotoxic phenotype. It is also remarkable that only a very small percentage of apoptotic cells was observed in all groups. In the islets of WT mice on HFD, there was a significant, almost 2.5-fold increase with respect to WT mice on CD (Figure 5b). This is less than the three-fold increase in apoptotic cells reported by Butler et al. (2003), but in that study, the apoptotic cells were evaluated in samples from obese, diabetic human patients [5]. The observed increases in apoptosis and, at the same time, in the number of beta cells in the islets of KO mice must be part of a cellular adaptation process to the altered glucose tolerance and insulin resistance. However, this difference cannot be taken to suggest the improved survival of mice in the long term under HFD conditions, since the lack of CAV1 has adverse effects on the entire metabolism, particularly in the adipose tissue and intracellular insulin signaling [33,38,41]. Further studies using animal models with a conditional KO of the CAV1 gene in beta cells would be necessary in order to define the specific role in vivo of CAV1 in beta cell survival under pro-diabetogenic conditions.

To date, several studies have reported that CAV1 induces sensitization to cell death in beta cells [53,54,55]. An association between CAV1 and MAPKs has been reported in various cell types. Knockdown of CAV1 diminishes the phosphorylation of p38 induced by heparin in murine peritoneal macrophages [56]. Additionally, CAV1 overexpression induced the activation of ERK, JNK and p38 signaling pathways in Hepa1-6 cells [57]. MAPKs have been associated with both anti- [58,59,60] and pro-apoptotic [18,19,20,21,22,23,61] effects in beta cells. Currently, the prevalent notion is that JNK and p38 activation induced by FFAs in beta cells is pro-apoptotic, while ERK1/2 activation is considered more anti-apoptotic [62]. In the present study, we observed significant activation of JNK, p38 and ERK in MIN6 cells after 6 h of incubation with palmitate (Figure 7). Notably, all MAPK inhibitors partially restored viability in MIN6 CAV1 cells exposed to palmitate (Figure 6c–e); however, the effect appeared more pronounced with the MEK/ERK inhibitor PD98059 (Figure 6e). Pro-apoptotic activity of ERK has been reported previously. Aberrant ERK activation by neurotoxic Aβ_42_ peptide leads to apoptosis in neurons [63], and prolonged activation of ERK beyond 24 and 48 h was associated with apoptosis induced by clove bud extracts in MCF-7 cells [64]. There are also reports showing that ERK activation by oxidative stress is associated with apoptosis in renal [65] and fibroblast cell lines [66]. Plaisance et al. showed in MIN6 cells that palmitate induces apoptosis in an ERK-C/EBP dependent manner [23]. Taken together, the available evidence suggests that in some cell types a stimulus that activates ERK signaling can induce cell death. In the MIN6 murine beta cell line, CAV1 silencing was associated with a reduction in ERK stimulation by insulin signaling in vitro and, in CAV1 KO mice, beta cells showed a significant decrease in ERK activation [16]. These results are consistent with the idea that CAV1 enhances ERK signaling.

Interestingly, in our study, all MAPK inhibitors tended to reduce the CAV1-induced sensitivity to palmitate (Figure 6c–e). However, pharmacological inhibitors are not entirely specific, and, particularly in the case of MAPK inhibitors, the problem of crosstalk between p38, JNK and ERK [67] complicates the interpretation of results. Experiments that involve loss-of-function through silencing with small interfering RNAs (siRNA) and gain-of-function upon overexpression of specific MAPKs in beta pancreatic cells exposed to saturated fatty acids are required in order to shed more light on the mechanisms implicated in beta cell responses to stress. For instance, ERK inhibition by p38 overexpression does not seem to be essential for stearate-induced apoptosis [68]. However, Wei et al. reported that inhibition of p38 improved beta cell function and reduced beta cell apoptosis in *db/db* mice [20]. Moreover, p38 MAPK seems to be one of the most important kinases involved in the induction of apoptosis by FFAs in pancreatic β-cells [62]. Thus, further studies involving the silencing and overexpression of specific MAPKs in islets and beta cells will be required in order to determine their precise role in beta cell death induced by saturated fatty acids.

## 4. Conclusions

In conclusion, our data suggest that CAV1 KO mice exhibit an altered metabolic state but that HFD does not significantly alter their metabolic condition, as might have been expected, presumably because their islets are still able to adapt to a HFD. This interpretation is supported by the observed changes in islet morphology and the decrease in beta cell apoptosis after long-term HFD exposure. These observations are in agreement with our previous report showing the in vitro effects of CAV1 absence in the MIN6 murine beta cell line exposed to palmitate [9]. However, considering that the lack of CAV1 in knock-out mice alters the lipid metabolism and insulin sensitivity, it is difficult to separate systemic effects from beta cell-specific effects due to CAV1 deletion. Future studies with animal models lacking the expression of CAV1 in a beta cell-specific manner will be necessary in order to elucidate more definitively the role of CAV1 in beta cell resistance and adaptation to lipotoxic conditions in vivo. On the other hand, our in vitro studies with the MIN6 murine beta cell line, as reported here, suggest that the sensitizing effects of CAV1 to palmitate are mediated, at least in part, by the increased activation of MAPKs, particularly ERKs. Further studies using loss- and gain-of-function strategies with these kinases will help us to determine the importance of the CAV1–MAPK connection in beta cell survival.

## 5. Material and Methods

### 5.1. Animals

Male C57Bl/6J mice of 8 weeks of age were used as wild type animals (WT) and were obtained from the animal facility of the Center for Studies on Exercise, Metabolism and Cancer (CEMC), Faculty of Medicine, University of Chile (Santiago, Chile) Male CAV1 KO mice (B6.CgCav1/J HOM, homozygous for CAV1<1tm 1 Mls; with C57BL/6J background, Jackson Laboratory) were kindly provided by Dr. Alvaro Lladser, Laboratory of Gene Immunotherapy of Fundación Ciencia & Vida (Santiago, Chile) Both WT and CAV1 KO mice were housed in the CEMC facility at a constant temperature of 21 °C, with a 12/12 h light/dark cycle. At 9 weeks of age, mice were divided into four groups of 7 mice each. Two groups, the WT and CAV1 KO control diet (CD) groups received a commercial control diet containing in kcal 14% fat, 26% protein and 60% carbohydrate (5P00 Prolab RMH 3000, LabDiet St. Louis, MO, USA). The other two WT and CAV1 high fat diet (HFD) groups received a commercial high fat diet containing in kcal 60% fat, 20% protein and 20% carbohydrate (D12492, Research diets, New Brunswick, NJ, USA). The animals were subjected to CD or HFD for 12 weeks, and then they were sacrificed and the epididymal fat was obtained. All of the animal procedures were approved by the Bioethics Committee of the Faculty of Medicine, Universidad de Chile (Project Number 1160257, Approved Protocol Nº CBA 0902 FMUCH, 2 December 2016.).

### 5.2. Intraperitoneal Glucose Tolerance Test (IPGTT)

At the end of the CD or HFD, WT and CAV1 KO mice were subjected to 6 h of fasting to avoid unnecessary catabolic stress and to assess the IPGTT within a physiological context [69]. Mice were weighed and 2 g of glucose per kilogram of weight in a calculated volume (volume of glucose injection in µL = 10 × body weight (g)) was infused intraperitoneally, and consecutive caudal capillary blood samples were taken after 0, 15, 30, 60 and 120 min. The Accutrend Glucose Test strips system (Roche Diagnostics, Indianapolis, IN, USA) was used to quantify blood glucose. The areas under the IPGTT curves (AUC) were calculated by using the GraphPad Prism 5 software.

### 5.3. Blood Samples

At the end of 12 weeks on CD or HFD, and one day after IPGTT, 6-h fasting mice were sacrificed, and blood samples were obtained by cardiac puncture with a heparinized syringe. The blood samples were centrifuged for 10 min at 2000× *g* in a refrigerated centrifuge to obtain plasma. The plasma samples were stored at −20 °C until glucose (GOD kit, HUMAN Diagnostics, Wiesbaden Germany), insulin (EZRMI-13K rat/mouse insulin ELISA, Merck, Darmstadt, Germany), total cholesterol (CHOL kit, HUMAN Diagnostics, Wiesbaden, Germany), triglycerides (TG kit, HUMAN Diagnostics, Wiesbaden, Germany), free fatty acids (EnzyChrom Free Fatty Acid Assay Kit, Universal Biologicals Ltd., London, UK), C-peptide (C-Peptide (human/mouse/rat) EIA Kit, Biovision, Milpitas, CA, USA), carbonylated protein (protein carbonyl colorimetric assay kit, Cayman Chemical, Ann Arbor, MI, USA) and total protein concentration (BCA assay, Pierce, Thermo Fisher Scientific, Waltham, MA, USA) were measured. The homeostasis model assessment of insulin resistance (HOMA-IR) was calculated from fasting blood glucose and insulin levels by using a modified version of the standardized equation for humans. The classical factor 22.5 was replaced by 31.1, a factor derived from the basal concentrations of glucose (median 6.9 mmol/L) and insulin (median 4.5 mU/mL) in WT mice, according to the following formula: (glucose (mmol/L) × insulin (mIU/L)/31.1. The hepatic insulin clearance was calculated from the basal insulin/C-peptide ratio, assuming that equimolar amounts of insulin and C-peptide are secreted and that insulin but not C-peptide is removed by the liver [70].

### 5.4. Islet Extraction and Study of Islet Morphology

The pancreas was extracted from male mice after 12 weeks on CD or HFD, and it was dissected and fixed in 10% p-formaldehyde in a neutral buffer solution overnight and embedded in paraffin (Electron Microscopy Science, Hatfield, PA, USA). Sections of 4 μm were deparaffinised, rehydrated and incubated with citrate buffer (10 mmol/L; pH 6.0) inside a microwave oven. Mouse pancreas sections were permeabilized with 0.1% Triton X-100 in PBS, rinsed with PBS and blocked for 1 h with PBS-1% Bovine Serum Albumin (BSA) at room temperature. Primary polyclonal antibodies against insulin (1/200, Polyclonal Guinea Pig Anti-Insulin A564, Dako Agilent, Santa Clara, CA, USA) and nuclear staining with DAPI (Tocris, Bioscience, Minneapolis, MN, USA) were used to visualize islets. As a secondary antibody to visualize insulin, an Alexa Fluor-633 goat anti-guinea pig antibody (1/200, Molecular Probes, Invitrogen, Carlsbad, CA, USA) was used. The samples were treated with Dako anti-fading reagent (Dako, Glostrup, Denmark) and stored at 4 °C. Images were converted to 8 bit and to binary colors before evaluation using the ImageJ program. Whole sections were stained for DAPI and insulin and then imaged as 10x tile scans on a C2 confocal microscope system (Nikon Instruments Inc., Minato, Tokyo, Japan). Islet size and circularity were determined using ImageJ on the insulin-stained sections of pancreas, counterstained with DAPI. The insulin-positive area for each islet was measured, and the number of nuclei (DAPI) present in the insulin-stained area (mm^2^) was manually counted in order to calculate the individual beta cell cross-sectional area (mm^2^). The percentage of beta cell area per islet area was calculated. The circularity of the islets was calculated with the ImageJ plugin using the following equation: circularity = 4π(area/perimeter^2^). Here, a value of 1.0 indicates a perfect circle, and as the value approaches 0.0, it becomes indicative of a more elongated shape [52].

### 5.5. Study of In Situ Islet Apoptosis

Pancreas sections obtained as described in Section 2.3 were used for the determination of islet apoptosis, assessing DNA fragmentation by the Terminal Deoxynucleotidyl Transferase dUTP Nick End Labeling (TUNEL) method, following the manufacturer’s instructions (Fluorometric DeadEndTM TUNEL System, Promega). In the same procedure, pancreas sections were stained with DAPI (Tocris, Bioscience, MN, USA), anti-mouse insulin antibody (1/200, Polyclonal Guinea Pig Anti-Insulin A564, Dako Agilent, Santa Clara, CA, USA) and Alexa Fluor 633 goat anti-guinea pig (Thermo Fisher Scientific) antibodies in order to identify and quantify the TUNEL-positive cells inside the islets using the ImageJ software. Samples were treated with Dako anti-fading reagent (Dako, DAgilent, Santa Clara, CA, USA) and stored at 4°C until their analysis by immunofluorescence microscopy.

### 5.6. Cell Culture

As a mouse beta pancreatic in vitro model, we used a MIN6 murine beta cell line lacking CAV1 [9] that was stably transfected with either the empty vector pLacIOP (MIN6 Mock) or pLacIOP-caveolin-1 (MIN6 CAV1) [71]. The cells were cultured in Dulbecco’s Modified Eagle’s Medium (DMEM) (Gibco, Invitrogen Carlsbad, CA, USA) high glucose (4.5 g glucose/L) supplemented with 10% of fetal calf serum (Biological Industries, Cromwell CT, USA) and antibiotics (10,000 U/mL penicillin and 10 µg/mL streptomycin, Biological Industries, Cromwell CT, USA) at 37 °C, in a humidified chamber with 5% CO_2_.

### 5.7. Free Fatty Acid Solutions

The fatty acids palmitate (sodium palmitate, Sigma-Aldrich, St. Louis, MO, USA) and oleate (Sigma Aldrich, St. Louis, MO, USA) were prepared by dissolving 100 mmol/L of palmitate or oleate in 0.1 M NaOH at 70 °C. Then, these solutions were diluted 1/20 in 10% BSA (fatty acid-free bovine albumin, Sigma Aldrich, St. Louis, MO, USA) at 55 °C and stirred for 10 min. These 10X stock solutions (5 mM of palmitate or oleate in BSA) were sterilized by ultrafiltration (Merck Millipore filter 0.22 µm), aliquoted and stored at −20 °C. Each aliquot was thawed only once. As a control, the vehicle containing NaOH alone with BSA was used.

### 5.8. MAPK Inhibitors

To evaluate the importance of the three major MAPKs in the sensitization to lipotoxicity induced by CAV1, we used the following compounds: SP600125 (JNK 1/2/3 inhibitor), SB204580 (p38 inhibitor) or PD98059 (MEK1/2 inhibitor to prevent ERK1/2 activation). The three inhibitors were purchased from Santa Cruz Biotechnology (Dallas, TX, USA). DMSO at a final concentration of 0.5% alone with culture medium was used a control. All the inhibitors were dissolved in a stock solution in DMSO (Duchefa Biochemie, Amsterdam, Netherlands).

### 5.9. Cell Viability Assays

The viability of MIN6 cells expressing or not expressing CAV1 in lipotoxic in vitro conditions was assessed using the MTT kit (Promega, Madison, WI, USA). In brief, MIN6 cells were seeded in 96-well plates for 24 h, and then different concentrations (0.25, 0.5 and 1.0 mM) of palmitate or oleate were added for 24 h. At the end of this period, the MTT reagent was applied, following the manufacturer’s instructions. In the same way, viability was assessed in cells pre-incubated with MAPK inhibitors.

### 5.10. Western Blotting

Cells were cultured up to 80% confluence and then washed twice with cold PBS and lysed in RIPA buffer (Sigma-Aldrich, St. Louis, MO, USA) containing 50 mM TRIS (Sigma-Aldrich St. Louis, MO, USA), 150 μM NaCl, 0.25% sodium deoxychloride, 1% NP40 and SDS (Sigma-Aldrich, St. Louis, MO, USA) 0.1%, plus a cocktail of protease/phosphatase inhibitors (100 μM EGTA, 0.5 M EDTA, 100 μM PMSF, 1 M Na_3_VO_4_, 0.5 M Na_2_P_2_O_7_, 1 M NaF) and a protease inhibitor cocktail (CalBiochem, Darmstadt, Germany), and they were then sonicated (Sonic Model 100; Fischer Scientific, Pittsburgh, USA). Protein concentrations were determined using the BCA assay (Pierce, ThermoFisher Scientific). Total protein extracts (30 μg per condition) were separated by SDS-PAGE (10% gel solution) using the Mini PROTEAN kit (BioRad, Philadelphia, PA, USA) and transferred to 0.45 µm NC nitrocellulose membrane (Mersham Protran, Mannheim, Germany) using the Mini Tansblot Kit (BioRad, Philadelphia, PA, USA). The membrane was blocked with 3% BSA in Tween/TBS 1X and labeled with anti-HSP90 α/β (F-8) antibodies (Santa Cruz Biotechnology, Dallas, TX, USA), anti-JNK antibody (D-2) (Santa Cruz Biotechnology, Dallas, TX, USA), anti-p-JNK antibody (14.Thr183 / Tyr185; (Santa Cruz Biotechnology, Dallas, TX, USA), anti-p38α/β antibody (A-12) (Santa Cruz Biotechnology, Dallas, TX, USA), anti-p-p38 (E-1 Tyr182); (Santa Cruz Biotechnology, Dallas, TX, USA), anti-ERK1/2 (E-12) (Santa Cruz Biotechnology, Dallas, TX, USA) or anti-p-ERK 1/2 antibody (12D4) (Santa Cruz Biotechnology, Dallas, TX, USA). Band intensities were quantified by scanning densitometry and processed using FIJI Software (www.fiji.sc) [72].

### 5.11. Statistical Analysis

In vivo results are expressed as individual points with their respective means. In vitro results are expressed as mean ± SD. For comparisons between WT and KO mice on different diets in in vivo studies, a two-way ANOVA was applied with a Bonferroni post-test. In the in vitro studies, for two group comparisons, the non-parametric Mann–Whitney test was applied. For multiple comparisons, the non-parametric Kruskal–Wallis test and the post-hoc Dunn´s test were employed. Statistical analyses were determined using GraphPad Prism 7 software (GraphPad Software, California, USA, www.graphpad.com). A value of *p* < 0.05 was considered statistically significant.
